# Acupuncture modulates the microbiota-gut-brain axis: a new strategy for Parkinson’s disease treatment

**DOI:** 10.3389/fnagi.2025.1640389

**Published:** 2025-08-07

**Authors:** Zimo Zang, Fang Yang, Liang Qu, Minghui Ge, Liang Tong, Lihui Xue, Xiuye Sun, Ying Hai

**Affiliations:** ^1^Department of Rehabilitation, The Second Affiliated Hospital of Liaoning University of Traditional Chinese Medicine, Shenyang, China; ^2^Department of Neurology, The Affiliated Hospital of Liaoning University of Traditional Chinese Medicine, Shenyang, China; ^3^Anshan Hospital of Traditional Chinese Medicine, Anshan, China

**Keywords:** microbiota-gut-brain axis, Parkinson’s disease, acupuncture, pathogenesis, strategy

## Abstract

Parkinson’s disease is a relatively common neurodegenerative disorder in clinical practice, and its prevalence is increasing worldwide. It not only causes patients to have movement disorders such as tremors and delayed initiation but also makes them suffer from olfactory disorders, gastrointestinal disorders, insomnia and other symptoms, which imposes a heavy burden on both patients and their families. In recent years, some scholars believe that the gut-brain axis may be the key to revealing the pathogenesis of Parkinson’s disease. The changes in intestinal flora, or bacterial infections and oxidative stress, lead to abnormal aggregation of alpha-synuclein and formation of neurotoxic Lewy bodies, which are transmitted to the central nervous system via the vagus nerve, thus causing Parkinson’s disease. A large number of evidence-based studies have shown that acupuncture is effective in treating motor disorders and non-motor symptoms such as constipation, neuropsychiatric symptoms, and dysphagia symptoms in Parkinson’s disease, also this treatment is safe. However, its mechanism remains unclear. Acupuncture may affect the gut-brain axis and treat PD by improving intestinal flora imbalance, interfering with the expression of alpha-synuclein protecting neurological function, reducing imflammation, and influencing glial cells, etc. Therefore, the aim of this review is to elucidate the pathogenesis of PD from the perspective of neural, immune, and metabolic signaling pathways of the microbiota-gut-brain axis. In addition, this paper integrates the mechanism of acupuncture treatment with the pathogenesis of PD for the first time and to provide potential new strategies for its treatment.

## 1 Introduction

Parkinson’s disease (PD) is the second most common neurodegenerative disease, mostly seen in middle-aged and older adults. Currently, there are approximately 6 million PD patients worldwide (Tolosa et al., 2021), with its incidence and prevalence continuing to increase (Bosch-Barceló et al., 2025). It has been estimated that by 2030, in China, this figure will be more than doubled, reaching between 8.7 million and 9.3 million (Dorsey et al., 2007). PD primarily affects patients’ motor function, with symptoms such as bradykinesia, resting tremor and ankylosis, while in the early stages of PD, patients usually present symptoms (insomnia, depression, and olfactory disturbances) (Shah et al., 2009), gastrointestinal (GI) dysfunction (nausea, abnormal salivation, constipation, prolonged intestinal transit time, etc.) (Mulak and Bonaz, 2015) and other non-motor symptoms.

The main pathological features of PD include the loss of dopaminergic neurons in the substantia nigra (SN) and the abnormal aggregation of alpha-synuclein (*α*-syn). It has been found out that the patients with PD often have long-term gastrointestinal symptoms, such as constipation and intestinal dysfunction, before the appearance of motor symptoms, suggesting that the intestine may be one of the early pathological starting points of PD (Mulak and Bonaz, 2015). Gastrointestinal dysfunction results mainly from the changes of intestinal flora. The gut-brain axis (GBA) is a bidirectional communication system between the enteric nervous system (ENS) and the central nervous system (CNS) of the brain through neural, endocrine, and immune pathways that regulate multiple physiological functions. Among them, gut mirobiota plays a key role in this interaction. Alterations in gut flora may lead to abnormal aggregation of *α*-syn (Wu J. et al., 2025). Abnormally aggregated α-syn spreads from the ENS to the CNS through the vagus nerve (VN) and is eventually transmitted to the SN, leading to the loss of dopaminergic neurons and ultimately PD (Del Tredici and Braak, 2016). Therefore, scientists have proposed the hypothesis that PD may cause changes in gastrointestinal function from the gut microbiota and transfer to the CNS via the GBA.

At present, the treatment of PD mainly relies on the drugs that increase dopamine concentrations or directly stimulate dopamine receptors (Kalia and Lang, 2015). However, during the treatment period, the drug often accelerates the degeneration of the substantia nigra pars compacta (SNpc), resulting in a gradual weakening of the drug’s efficacy and aggravating motor disorders and dystonia for the patient (Lipski et al., 2011). Meanwhile, most PD patients have GI symptoms such as gastroparesis and dysphagia, which further impede drug absorption. Additionally, some of the anti-Parkinson’s disease medications may exacerbate the GI symptoms in PD patients (Sung et al., 2014). Therefore, there is an urgent need to develop safer and more effective solutions to the treatment of PD. As a kind of external operation method based on the main principle of traditional Chinese medicine, acupuncture activates the body’s inherent regulating ability and exerts a multi-pathway, multi-target regulating effect (Wu Y. et al., 2025). Moreover, a large number of evidence-based studies have shown that acupuncture can significantly improve motor disorders and non-motor symptoms such as constipation, neuropsychiatric, and dysphagia in PD patients (Cao et al., 2020). As a supplementary and alternative therapy, acupuncture has received increasing attention and research (Jiang et al., 2018; Wen et al., 2021). In summary, this review aims to clarify the pathogenesis of PD from the perspective of neural, immune, and metabolic signaling pathways of the microbiota-gut-brain axis. On this basis, this review discusses the integrative application of acupuncture in the treatment of Parkinson’s disease and its possible mechanisms, aiming to provide potential new strategies for its treatment.

## 2 Altered gut-microbiota present in PD patients

The intestines contain nearly 100 trillion microorganisms (Vilela et al., 2024). Specifically, the microbiota includes bacteria, eukaryotes, viruses, archaea, fungi and protozoa (Cox et al., 2013). The microbiota collectively encode more than 3.3 million non-redundant genes (more than 150 times the number encoded by the human host genome), and many microbial gene products have important implications for host metabolism and health (Collins et al., 2012). Meta-analysis of the intestinal microbiome in PD via 16S rRNA gene sequencing and shotgun metagenomics showed that the abundance of *Streptococcus, Bifidobacterium, Lactobacillus, Akkermannia,* and *Desulfovibrio* was increased, whereas the abundance of *Roseburia*, *Faecalibacterium*, *Blautia*, *Lachnospira* and *Prevotella* was decreased in PD patients (Nie et al., 2022). Another meta-analysis of 21 controlled studies of PD patients and normal subjects targeting the PD gut microbiome showed that, compared to healthy controls, two phyla (*Synergistetes* and *Verrucomicrobia*) and five families (*Synergistaceae, Verrucomicrobiaceae, Peptococcaceae., Clostridiales Incertae Sedis XII* and *Porphyromonadaceae*) and four genera (Eisenbergiella, Akkermansia, Desulfurispora and Acidaminobacter) increased, while one family (*Lachnospiraceae*) and two genera (*Faecalibacterium* and *Roseburia*) decreased (Kleine Bardenhorst et al., 2023). Bai et al. (2024). reported an increase in *Bifidobacteriaceae*, *Ruminococcaceae*, *Rikenellaceae, Lactobacillaceae, Verrucom icrobiaceae* and *Christensenellaceae* in patients with PD, whereas *Prevotellaceae, Lachnospiraceae, Erysipelotrichaceae* and *Faecalibacterium* decreased. Shen et al. (2021) also conducted a meta-analysis of PD patients and healthy control studies of the gut microbiome of PD, which showed that PD patient *Bifidobacteriaceae, Ruminococcaceae, Verrucomicrobiaceae* and *Christensenellaceae* were elevated and *Prevotellaceae, Faecalibacterium* and *Lachnospiraceae* were decreased compared to healthy controls. Romano et al. also reported a meta-analysis of the PD gut microbiome involving 22 studies, which showed that *Lactobacillus, Akkermansia, Hungatella,* and *Bifidobacterium* were elevated in patients with PD, whereas *Roseburia, Fusicatenibacter Blautia, Anaerostipes (Trichosporonaceae)* and *Faecalibacterium (Ruminococcaceae)* were decreased in patients with PD (Romano et al., 2021). By summarizing the above meta-analyses, it was found that *Verrucomicrobiaceae Akkermansia* were increased and *Roseburia, Faecalibacterium* and *Prevotellaceae* was decreased in patients with PD. Zhu et al. (2022) also have summarized that most gut microbiota of PD patients shown PD-related changes in the gut microbial composition, including increases in the relative abundance of *Verrucomicrobiaceae* and *Akkermansia* and decreases in *Prevotellaceae* and *Prevotella*. These studies consistently report alterations in the gut microbiome of PD patients (Please refer to Table 1 for details).

**Table 1:** Changes in the intestinal microbiota.

Author	Number of studies	Increasing microbiota	Decreasing microbiota
Nie et al. (2022)	11 items	*Streptococcus, Bifidobacterium, Lactobacillus, Akkermannia,* and *Desulfovibrio*	*Roseburia, Faecalibacterium, Blautia, Lachnospira* and *Prevotella*
Kleine Bardenhorst et al. (2023)	21 items	two phyla (*Synergistetes* and *Verrucomicrobia*) and five families (*Synergistaceae, Verrucomicrobiaceae, Peptococcaceae., Clostridiales Incertae Sedis XII* and *Porphyromonadaceae*) and four genera (Eisenbergiella, Akkermansia, Desulfurispora and Acidaminobacter)	one family (*Lachnospiraceae*) and two genera (*Faecalibacterium* and *Roseburia*)
Bai et al. (2024)	14 items	*Bifidobacteriaceae*, *Ruminococcaceae*, *Rikenellaceae, Lactobacillaceae, Verrucomicrobiaceae* and *Christensenellaceae*	*Prevotellaceae, Lachnospiraceae, Erysipelotrichaceae* and *Faecalibacterium*
Shen et al. (2021)	14 items	*Bifidobacteriaceae, Ruminococcaceae, Verrucomicrobiaceae* and *Christensenellaceae*	*Prevotellaceae, Faecalibacterium* and *Lachnospiraceae*
Romano et al. (2021)	22 items	*Lactobacillus, Akkermansia, Hungatella,* and *Bifidobacterium*	*Roseburia, Fusicatenibacter Blautia, Anaerostipes (Trichosporonaceae)* and *Faecalibacterium (Ruminococcaceae)*

In addition, the gut microbiota can influence the ENS homeostasis. The ENS is a large branch of the autonomic nervous system. Known as the “second brain,” it is highly similar to the CNS. The ENS consists of more than 100 million neurons and more than 400 million enteric glial cells (EGCs) that coordinate and regulate gastrointestinal functions (Dowling et al., 2022). EGCs are a group of peripheral glial cells associated with the somata and synapses of intestinal neurons, existing at all extensions of the gastrointestinal tract. They play an important role in the secretion and absorption of the intestinal epithelium (Montalbán-Rodríguez et al., 2024). Enterochromaffin cells (ECs) and enteroendocrine cells (EECs) located in the intestinal epithelium, make contact directly with approximately 4 trillion gut microbes (Dicks, 2023). Thus, gut microbiota influence the ENS homeostasis through the EECs. Secondly, the gut microbiota maintains ENS homeostasis by regulating enteric neurons and the EGCs. A recent study reported that gut microbiota was critical for maintaining the integrity of the ENS by regulating enteric neuron survival and promoting neurogenesis (Vicentini et al., 2021). Kabouridis et al. (2015) found that the EGC is a continually renewed homeostatic cell population in adult mice, whose homeostasis and development are regulated by gut flora, which in turn affects the ENS homeostasis.

Dysbiosis of gut microbiota has also been regarded as a trigger of or contributor to PD. Yu et al. (2023) demonstrated that dysbiosis of gut microbiota promoted neurobehavioral deficits and oxidative stress responses in a rat model of PD. Changes in intestinal flora can improve symptoms of PD. Wang et al. (2021) confirmed that the brain dopa/dopamine levels of PD model mice could be elevated by regulating the intestinal flora, thus achieving the therapeutic purpose. In addition, the increased abundance of *Muribaculaceae, Lactobacillaceae, Lachnospiraceae,* and *Eggerthellaceae*, as well as depletion of the abundance of *Aerococcaceae*, and *Staphylococcaceae* can also lead to a reduction in motor symptoms and alleviation of neuroinflammation in PD mice (Cui et al., 2022). A clinical study demonstrated that fecal microbiota transplantation (FMT) could significantly improve the quality of life for PD patients by restoring their intestinal ecosystem (Cheng et al., 2023). Numerous clinical studies have shown that probiotics can improve the symptoms of constipation (Hong et al., 2022) and dyskinesia (Chu et al., 2023) in PD patients.

## 3 The role of gut-brain axis signaling pathways in Parkinson’s disease

### 3.1 Metabolic signaling pathway-short chain fatty acids

Short-chain fatty acids (SCFAs), such as butyrate, acetate, lactate, and propionate, are mainly produced by dietary fiber through fermentation in the colon by intestinal flora such as *Bifidobacteria*, *Lactobacillus* and *Trichoderma reesei*, providing energy to epithelial cells (Cushing et al., 2015). They can directly cross the blood-brain barrier (BBB), affecting on the CNS (Kalyanaraman et al., 2024). It is currently known that PD patients have reduced levels and diversity of gut flora producing SCFAs, especially propionate and butyrate (Kalyanaraman et al., 2024). Serum SCFAs are altered, while reduced serum propionic acid levels are correlated with motor symptoms, cognitive performance, and non-depressive states in PD patients (Wu et al., 2022). Moreover, the ability to produce SCFAs after fiber fermentation *in vitro* fecal in PD patients declines compared to healthy individuals (Baert et al., 2021).

SCFAs play an important role in maintaining homeostasis of the GBA. First of all, SCFAs enhance the intestinal mucosal barrier. Huang et al. used propionate to produce beneficial effects on the intestinal epithelial barrier and improve locomotor function in mice with 1-methyl-4-phenyl-1,2,3,6-tetrahydropyridine (MTPT) induced PD (Huang et al., 2021). Kelly et al. enhanced the tissue barrier function through crosstalk between SCFAs and intestinal epithelial HIF (Kelly et al., 2015). Moreover, SCFAs may further prevent bacteria and bacterial products from entry into the systemic circulation by enhancing the mucosal barrier. A recent experiment has shown that butyrate reduces barrier disruption by decreasing the local inflammatory response and also has a direct protective effect on cytokine-mediated barrier disruption (Korsten et al., 2023). Secondly, SCFAs also contribute to maintaining the integrity of BBB. Mei et al. significantly enhanced BBB integrity in rhesus monkeys suffering from intestinal dysbiosis by means of supplementation with SCFAs (Chenghan et al., 2025). Wang l. et al. (2023) used inulin to enhance intestinal mucosal integrity, causing an increase in SCFAs, which in turn improved depressive behavior, BBB integrity, and neuroinflammation in mice. SCFAs also contribute to attenuating neuroinflammation and affect microglia maturation (Erny et al., 2015). A recent experiment also demonstrated that the SCFAs suppressed MPTP-induced neuroinflammation in PD mice, entered the SNpc, and inhibited dopamine neurons, thereby alleviating dyskinesia in PD mice (Hou et al., 2021a). Meanwhile, SCFAs also reduce microglia and astrocyte activation (Soliman et al., 2013; Patnala et al., 2017), inhibit inflammatory responses induced by lipopolysaccharides (LPS) (Wang et al., 2018) and reduce the expression of pro-inflammatory factors such as IL-1β, IL-6, and others (Soliman et al., 2012). Thirdly, SCFAs can reduce *α*-syn expression. A recent study has shown that neuronal α-syn aggregation induces a mitochondrial unfolded protein response (mitoUPR) in the gut, leading to reduced levels of propionate. A short-chain fatty acid propionate may prevent α-synuclein-induced neuronal death and locomotor deficits through bidirectional regulation of the intestinal and neuronal processes in a cryptic nematode model of PD (Wang et al., 2024). Another study also showed that SCFAs reduced the accumulation of *α*-syn in the colon and hippocampus of PD mice, prevented dopaminergic cell death in the substantia nigra, and improved motor and non-motor symptoms (Techaniyom et al., 2024). Finally, SCFAs are also significantly associated with neurotransmitter concentrations. The HDAC-inhibitory behavior of sodium butyrate has been shown to exert beneficial effects on neurotoxicity-induced rats by improving motor symptoms and increasing striatal dopamine (Sharma et al., 2015). The HDAC inhibitory behavior of sodium butyrate has been demonstrated to have beneficial effects on neurotoxic-induced rats by improving motor symptoms and increasing striatal dopamine (Paiva et al., 2017). Meanwhile, it has been shown that the microbial metabolite SCFAs, especially butyrate, which can be transported into the circulation, also increase brain 5-hydroxytryptamine concentration and exert neuroprotective effects in mice (Sun et al., 2016).

Thus, there are beneficial effects of SCFAs on both the neurological and immune systems of PD patients (Kalyanaraman et al., 2024). SCFAs may improve PD symptoms by reinforcing the intestinal mucosal barrier, further preventing the entry of bacteria and bacterial products into the body’s circulation system, maintaining the integrity of the BBB, attenuating neuro inflammation, influencing microglia maturation, reducing the activation of microglia and astrocytes, decreasing the expression of *α*-syn, and increasing the concentration of neurotransmitters such as 5-HT.

### 3.2 Neural signaling pathway

#### 3.2.1 Vagal transmission of alpha-synuclein

α-syn is a naturally derived protein that abundantly present in healthy neuronal cells (Mahbub et al., 2024). Under normal state, the tendency of α-syn aggregation is relatively low (Lashuel et al., 2013). Abnormal aggregation of α-syn occurs in the gut due to disturbed gut microbiota in PD patients (Goya et al., 2020; Fang et al., 2024). *α*-Syn spreads between neurons like a prion virus, which leads to more monomeric α-syn misfolding or over-modification. Then, it is deposited in neurons to form Lewy bodies (LBs), resulting in neurotoxicity (Bu et al., 2020). Braak et al. (2006) proposed a hypothesis in 2003 that α-syn transmits through the VN and dorsal motor nucleus of the vagus nerve (DMV) in the medulla oblongata from the ENS to the CNS.

Firstly, gut microbiota can affect *α*-syn. A recent meta-analysis revealed that mucin-degrading *Akkermansia* is increased and the bacteria produced by short-chain fatty acid (SCFAs) are reduced in PD. These changes in gut flora leads to increase intestinal permeability and exposure of the intestinal plexus to toxins, such as LPS, which can lead to aberrant *α*-syn aggregation (Hirayama and Ohno, 2021). Some gut microbiota themselves can also cause abnormal aggregation of α-syn (Sampson et al., 2020). Subsequently, abnormally aggregated α-syn can come into contact with the EGCs of neurons and ENS via the EECs located in the intestinal epithelium and spread the α-syn folding through neuro-foot junctions (Claudino Dos Santos et al., 2023). Synaptic nucleoprotein pathology and EGC proliferation have been found in the duodenum of patients with PD, and abnormal aggregation of α-syn induces reactive EGC proliferation, which in turn leads to the changes in ENS neurons (Emmi et al., 2023). Secondly, the alterations in ENS neurons and EGCs themselves can initiate α-syn misfolding (Montalbán-Rodríguez et al., 2024). The ENS may be one of the main sites of action for LBs in PD (Mirzaei et al., 2021).

The vagus nerve is the longest and the most widely distributed autonomic nerve, originating in the brainstem and traveling down through the neck and into the chest and abdomen. It holds motor and sensory information and provides neural innervation for multiple systems. Containing approximately 80% afferent fibers and 20% efferent fibers, the afferent nerves are responsible for transmitting taste, visceral, and somatic sensations. Also, the efferent fibers are involved in the regulation of gastrointestinal, cardiac, and pulmonary functions (Han et al., 2022). In the gut, the VN afferent nerves terminate in the muscularis propria and mucosa. In the muscularis propria, vagal afferent nerves form intranodal endings and intramuscular arrays. In contrast, some vagal endings synapses, originating from the neurons, are connected to the enteric nervous system (Fülling et al., 2019). Moreover, the neurons of the ENS with the EGC itself originate from the vagus nerve and sacral neural crest (Natale et al., 2021). Thus, it is through the VN via the ENS that α-syn diffuses to the CNS.

The animal experiments performed by Holmqvist et al. (2014) demonstrated that α-syn folds misfold, deposit in the ENS and are transported to the brain via the VN. Vagotomy has been proven to reduce the risk of PD, further supporting the possibility that α-syn may spread along the vagus nerve to the central nervous system (Borghammer and Hamani, 2017). A systematic review of vagal ultrasonography for patients with PD indicates that PD patients have a certain degree of the VN atrophy (Abdelnaby et al., 2021). According to the hypothesis proposed by Braak, the aggregated α-syn diffuses from the ENS to the CNS via the vagus nerve, forming LBs and Lewy synapses (Forno, 1987). He divided the pathologic staging of PD disease into six stages (Braak et al., 2003). Firstly, the LBs are confined to the medulla oblongata, with the IX/X dorsal motor nuclei. As the condition gradually worsens, it affects the lower and upper brainstem or initially involves the anterior medial temporal mesocortex and eventually severely involves the brain, including neocortical areas. This, in turn, damages the substantia nigra and even the neocortical regions. The LBs of ENS can be taken as a pathophysiologic correlate of gastrointestinal signs in patients with PD.

The VN stimulation can also alleviate the symptoms of PD. A clinical trial conducted by Zhang et al. demonstrated that transcutaneous auricular vagus nerve stimulation (taVNS) relieved gait disturbances and remodeled sensory-motor integration in patients with PD (Zhang et al., 2023). A recent animal study also indicated that mild to moderate intensity vagus nerve stimulation (VNS) had a significant effect on 6- OHDA administration and exerted anti-inflammatory and neuroprotective effects on a rat model of PD induced by 6- OHDA administration (Kin et al., 2021).

#### 3.2.2 Regulation of neurotransmitters

##### 3.2.2.1 Dopamine

Dopamine (DA) is a central pathogenetic factor in PD, and its deficiency leads to dysfunction of the substantia nigra-striatal pathway, which in turn causes the motor symptoms of PD (Antonelli and Strafella, 2014). DA is synthesized mainly in neuronal cells of the brain and is largely dependent on Tyrosine, which is catalyzed by Tyrosine Hydroxylase (TH) to produce Levodopa (L-DOPA). Then, it is converted to DA by DOPA Decarboxylase (DDC). Tyrosine is mainly derived from the liver, kidney or diet, with the subsequent synthesis accomplished in the brain by tyrosine hydroxylase (TH) and DDC (Wang et al., 2021). DA levels are directly influenced by gut microbiota. The acetogens *E. limosum* and *B. producta* can synthesize DA via O demethylation of 3MT (Rich et al., 2022). For decades, L-dopa has been used to treat symptoms caused by the depletion of endogenous DA in the brains of patients with PD (Hauser, 2009). However, l-dopa can be metabolized by the intestinal microbiota, thus affecting its efficacy (Sandler et al., 1971). The presence of fecal commensal enterococci in the gut microbiota of PD patients is associated with increased l-dopa metabolism in the gut microbiota (Jameson and Hsiao, 2019). Thus, gut microbiota can modulate L-dopa content in humans, which affects its bioavailability, and thus affecting DA content and improving PD symptoms.

##### 3.2.2.2 Hydroxytryptamine

Hydroxytryptamine (5-HT), also known as serotonin, is an important neurotransmitter in the CNS for regulating mood, sleep and appetite (Pourhamzeh et al., 2022). A clinical trial indicated that the decreased platelet 5-HT levels are associated with resting tremor in PD, suggesting that 5-hydroxytryptaminergic disorders are involved in resting tremor in PD (Wang J. Y. et al., 2023). The 5-HT system originates from the nucleus of the middle suture and projects to the BG (basal ganglia), including the SNpc and caudate nucleus (Jacobs and Azmitia, 1992). In patients with PD, 5-HT neurotransmission is decreased in the late stage of the disease due to degeneration of dorsal nucleus of the middle suture (Jellinger, 2015). It is evidenced that 5-HT plays an important role in modulating mood, cognitive, and motor deficits in PD patients. As a precursor for serotonin biosynthesis, tryptophan crosses the blood–brain barrier and then getsmetabolized to serotonin in the nucleus accumbens within the brainstem (Le Floc’h et al., 2011). It is now known that serotonin synthesis occurs in ECs and ENS in the gastrointestinal tract (Mawe and Hoffman, 2013), ECs and enteric neurons catalyze the formation of 5-HT precursors from tryptophan via tryptophan hydroxylase (TPH), which is subsequently decarboxylated to form 5-HT. It is present in the gastrointestinal tract in approximately 95% of cases. TPH is divided into two isoforms, namely TPH1 and TPH2, with TPH1 predominantly found in the EC and TPH2 expressed in the CNS and enteric neurons (Sancho-Alonso et al., 2024). Previous studies have shown that commensal bacteria (particularly spore-forming bacteria of the mouse and human microbiota) promote 5-hydroxytryptamine biosynthesis in colonic EC through a metabolite/cellular component-dependent mechanism (Yano et al., 2015). Its effects have been discovered in sterile mice, which exhibit impaired 5-HT production in the colon (but not in the small intestine) and low blood 5-HT concentrations. While symbiotic microbiota can synthesize serotonin directly from luminal tryptophan. Several bacteria belonging to Lactococcus, Lactobacillus, Streptococcus, *Escherichia coli* and Klebsiella have been reported as capable to produce serotonin by expressing tryptophan synthase (Gao et al., 2020). It is confirmed that altering the microbiota can improve the symptoms of 5-HT-related diseases. Also, 5-HT stimulates the growth of *Enterococcus faecalis*, *Escherichia coli*, and *Rhodococcus erythropolis* in cultures (Yano et al., 2015). Thus, bidirectional regulation can occur between gut microbiota and 5-HT.

### 3.3 Immune signaling pathway

The gut microbiota influences brain function by maintaining homeostasis of innate and adaptive immunity and by limiting acute and chronic inflammation in the gut and CNS (Bostick et al., 2022). This is due to the fact that PD patients have an altered gut. Through a rat model of PD, Koutzoumis et al. (2020) discovered that antibiotics reduced the diversity of the gut flora and the expression of pro-inflammatory cytokines in the rat vegetative striatum, thus reducing dopamine neuron loss and an improvement in motor deficits. Due to the overgrowth of Enterobacteriaceae in the intestinal tract, LPS titration is enhanced (Forsyth et al., 2011). In normal mucosa, bacteria and other nodules are confined to the intestinal lumen. However, in the gut of patients with PD, EGC is activated due to bacterial infection, oxidative stress, etc. Also, a further release of pro-inflammatory factors by EGC activation disrupts the ENS homeostasis (Seguella et al., 2020), leading to increased intestinal permeability and the formation of a “leaky gut” (Di Vincenzo et al., 2024). Due to loss of intestinal epithelial membrane integrity after leaky gut and entry of bacterial products (e.g., LPS) into the circulation, pro-inflammatory factors in the blood increase and activation of the peripheral immune system occurs (La Vitola et al., 2021; Di Vincenzo et al., 2024). Previous meta-analyses have pointed out that peripheral blood concentrations of pro-inflammatory cytokines such as IL-6, TNF-*α*, IL-2, IL-10, IL-1β, and C-reactive protein (CRP) are significantly elevated in patients with PD compared with healthy individuals (Qin et al., 2016). EGC persistent neuroinflammation increases the expression of misfolded α-synuclein within the ENS, which spreads centrally (La Vitola et al., 2021; Di Vincenzo et al., 2024).

Habitually up-regulationed pro-inflammatory cytokines enter the brain by the blood–brain barrier, leading to activation of microglia and astrocytes (Fernandez et al., 2019). Consequently, the BBB is disrupted (Lan et al., 2022; Warren et al., 2024), which in turn results in the opening of the CNS to peripheral vascular factors and immune factors (Lau et al., 2024). Also, α-syn can cross the attenuated BBB mediated by astrocytes, thus causing PD (Sheng et al., 2020). The study suggests that patients with PD do have a BBB leakage (Al-Bachari et al., 2020). An autopsy of patients with PD showed diminished BBB integrity in the striatum of patients with PD, which in turn led to SNpc dopamine loss (Gray and Woulfe, 2015). Microglia are subjected to sustained inflammatory response stimulation, formation of eosinophilic LBs in the cytoplasm due to abnormal levels of α-syn aggregates in SpNc, and mitochondrial dysfunction in DA neurons that leads to increased oxidative stress (OS), which triggers apoptosis. Also, OS promotes α-syn aggregation as well, forming a positive feedback loop (Lei et al., 2021).

## 4 Acupuncture intervention strategies

### 4.1 Acupuncture is a peripheral intervention therapy with potential for Parkinson’s treatment

The meta-analysis of acupuncture for the treatment of PD showed that acupuncture holds promise for the treatment of PD (Xue et al., 2024), with a high safety profile (Noh et al., 2017; Hou et al., 2021b).

First of all, acupuncture improves movement disorders in PD patients. Lei et al. conducted a meta-analysis of clinical studies on the treatment of motor symptoms in PD patients using acupuncture. The article included 268 documents, involving 16 studies and a total of 462 PD patients. However, there was a high risk of bias in the implementation of blinding. The study revealed that a certain dose must be reached for acupuncture treatment to achieve better therapeutic effects, but excessive acupuncture stimulation may cause the body to develop a certain degree of tolerance (Lei et al., 2023). Acupuncture of ST34 (Liangqiu), BL57 (Chengshan), HT3 (Shaohai), HT7 (Shenmen), KI3 (Taixi), KI7 (Fuliu), and SP4 (Gongsun) improved gait disorders and balance disorders in PD patients (Pereira et al., 2021). It’s a non-blinded study with a small sample size, and it is limited to analyzing the acute effect of one acupuncture treatment. Acupuncture combined with bee venom acupuncture improved motor deficits, including postural instability, gait disturbance, and gait speed in patients with PD compared with sham acupuncture (Cho et al., 2018). According to the results of two studies as mentioned above, acupuncture could significantly improve the motor functions of PD patients, but there is no significant difference relative to the comparison group.

Secondly, acupuncture can improve constipation symptoms in PD patients. Zhao et al. searched the Cochrane Central Register of Controlled Trials and multiple databases, including Embase and PubMed, with 11 studies and 960 patients involved. Three of the studies had a high risk of bias, but all of the studies were conducted in China, posing a risk of regional bias. The results indicated that acupuncture increased the number of spontaneous bowel movements in PD patients, improved quality of life, increased rectal resting pressure, and reduced the severity of chronic constipation (Zhao et al., 2024). Li Y. k. et al. (2023) reported that compared with the sham acupuncture group, acupuncture treatment increased the number of spontaneous bowel movements and had a more sustained efficacy. Another multicenter randomized controlled study demonstrated that electroacupuncture of ST25 (Tianshu), SP14 (Fujie), and ST37 (Shangjiuxu) points combined with conventional medication could be effective in treating PDC (Li K. et al., 2023). However, the follow-up period was not long enough and the patients were not blinded in the study, which may affect the test results.

Moreover, acupuncture may improve neuropsychiatric symptoms in patients with PD. Tan et al. searched eight databases, including PubMed, Embase, and CNKI, and included 28 studies with a total of 2,148 participants after screening. Most studies had a high risk of bias in the implementation of blinding, but all studies had relatively complete data, which reduced the risk of attrition bias and reporting bias. The results indicated that acupuncture may improve symptoms of depression, anxiety, cognitive dysfunction, impulse control disorders, and quality of life in patients with Parkinson’s disease in the short term (Tan et al., 2024). Mi et al. (2024) searched eight databases, including PubMed, the Cochrane Central Register of Controlled Trials, and CNKI, and ultimately included 15 studies involving 957 participants. Among them, 11 RCTs had a high risk of bias in the implementation of blinding, which may be related to the need for patients to report sleep outcomes subjectively. The results indicated that acupuncture can serve as an adjunctive treatment for sleep disorders shown in Parkinson’s disease. A clinical trial conducted by Fan et al. demonstrated that acupuncture of GV24 (Shenting), GV29 (Yintang), bilateral HT7 (Shenmen), bilateral SP6 (Sanyinjiao), and four divine acupuncture needles for 8 weeks significantly improved patients’ anxiety symptoms (Fan et al., 2022). Notably, this was the first randomized clinical trial of the effectiveness of an acupuncture treatment regimen targeted for anxiety in patients with PD. However, the only drawback is that all the participants were Chinese and there was probably some bias in using HAM-A score of at least 14 as the standard for evaluating anxiety in PD. Yan et al. conducted a clinical trial study on sleep quality of patients with PD, with the result showing that acupuncture on the four divine acupuncture needles, GV24 (Shenting), GV29 (Yintang), LI4 (Hegu), LR3 (Taichong), SP6 (Sanyinjiao), HT7 (Shenmen), ST36 (Zusanli)、BL62 (BL62) and KI6 (Zhaohai) significantly improved patients’ sleep disorders and even their quality of life (Yan et al., 2024). The test data were true and reliable while the outcome measurements were comprehensive. However, the follow-up period was limited to 4 weeks, and all the participants were Chinese.

Finally, acupuncture may also improve dysphagia in patients with PD. Wu et al. searched seven databases, including PubMed, Cochrane Library, and CNKI. Also, 10 RCTs with a total of 724 participants were included. However, most of the studies had a high risk of bias in terms of blinding, with all of the included studies conducted in China, indicating a regional bias. The results showed that acupuncture not only improved the swallowing function of PD patients, but also improved their nutritional status and reduced the incidence of pneumonia (Wu et al., 2023). A randomized controlled study showed that conventional medication combined with acupuncture significantly improved dysphagia symptoms and nutritional status in patients with PD (Zeng et al., 2025). Fukuda et al. also confirmed that acupuncture points ST36 (Zusanli), SP6 (Sanyinjiao), and LI4 (Hegu) increased swallowing-related tongue pressure and improved dysphagia in patients (Fukuda et al., 2016). According to the results of two studies as mentioned above, acupuncture could significantly improve the swallowing function of PD patients, but there is no significant difference relative to the comparison group.

In conclusion, acupuncture has high efficacy in improving non-motor symptoms such as constipation, neuropsychiatric symptoms in PD patients, which is worthy of clinical promotion (Please refer to Table 2 for details).

**Table 2:** Clinical trial.

Study	Sample size	Design	Acupoint	Endpoints	Effect size	Safety
Pereira et al. (2021)	7	Controlled crossover study	ST34 (Liangqiu), BL57 (Chengshan), HT3 (Shaohai), HT7 (Shenmen), KI3 (Taixi), KI7 (Fuliu), and SP4 (Gongsun)	Balance and gait parameters(gait speed, gait cadence, support base width,vertical trunk oscillation,left–right, trunk oscillation)	Gait speed, SMD 0.66, 95% CI [-0.43, 1.74] gait cadence, SMD 0.97, 95% CI [-0.16, 2.10] Support base width, SMD 0.25, 95% CI [-0.80, 1.30] Vertical trunk oscillation, SMD 0.66, 95% CI [-0.43, 1.75] Cleft-right, trunk oscillation, SMD 0.78, 95% CI [-0.32, 1.88]	No side effects of any treatment were reported
Cho et al. (2018)	Active treatment group, *n* = 24 The sham treatment group, *n* = 24 The conventional treatment group, *n* = 15	A single center, double-blind, three-armed randomized controlled trial	bilateral GB20, LI11, GB34, ST36, andLR3	Unified Parkinson’s Disease Rating Scale (UPDRS) part II and part III score	Active treatment group vs. the sham treatment group, SMD 0.12, 95% CI [-0.45, 0.68] Active treatment group vs. the conventional treatment group, SMD 1.05, 95% CI [0.36, 1.74] The sham treatment group vs. the conventional treatment group, SMD 0.73, 95% CI [0.06, 1.40]	No serious adverse events were noted during the study period
Li Y. J. et al. (2023)	Manual acupuncture, *n* = 39 Sham acupuncture groups, *n* = 39	A single center, single-blind, randomized controlled trial	Sishenzhen (four acupoints, consisting of GV21, GV19, and next to GV20 1.5 cun bilateral), GV24 (Shenting), GV29 (Yintang), ST25 (Tianshu), CV4 (Guanyuan), and ST37 (Shangjuxu)	complete spontaneous bowel movements (CSBM) score	Posttreatment, SMD 0.86, 95% CI [0.40, 1.33] Follow-up, SMD 0.73, 95% CI [0.27, 1.19]	No serious adverse events
Li K. et al. (2023)	Electroacupuncture group, *n* = 83 Waitlist control group, *n* = 83	Multi-centre, randomised, assessor-blinded trial	Qianding (GV21) to Xuanlu (GB5), Connect Qianshencong (EX-HN1) to Xuanli (GB6), Quchi (LI11), Hegu (LI4), Yanglingquan (GB34), Zusanli (ST36), Sanyinjiao (SP6), Taixi (KI3) and Taichong (LR3), Tianshu (ST25), Fujie (SP14), Shangjuxu (ST37)	Unified Parkinson’s Disease Rating Scale (UPDRS) score	SMD 1.02, 95% CI [0.70, 1.35]	The incidence of adverse events was modest, and no participant dropped out of the study due to acupuncture-related ill effects
Fan et al. (2022)	Real acupuncture, *n* = 32 Sham acupuncture, *n* = 32	A single center, double-blind, randomized controlled trial	GV 24 (shen ting), GV 29 (yin tang), bilateral HT7 (shen men), bilateral SP 6 (san yin jiao), and Si Shen Zhen	Hamilton Anxiety Scale (HAM-A) score	Posttreatment, SMD 0.12, 95% CI [-0.37, 0.61] Follow-up Secondary outcome, SMD 3.76, 95% CI [2.93, 4.59]	No serious adverse events occurred.
Yan et al. (2024)	Real acupuncture, *n* = 40 Sham acupuncture, *n* = 38	A single center, double-blind, randomized controlled trial	Si Shenzhen, ShenTing (GV24), YinTang (GV29), HeGu (LI4), TaiChong (LR3), SanYinJiao (SP6), ShenMen (HT7), ZuSanLi (ST36), ShenMai (BL62), and ZhaoHai (KI6)	Parkinson Disease Sleep Scale (PDSS) score	Posttreatment, SMD 1.18, 95% CI [0.70, 1.66] Follow-up, SMD 1.21, 95% CI [0.72, 1.69]	No participants withdrew from the study because of an AE.
(Zeng et al. (2025))	Experimental group, n = 56 Control group, *n* = 56	A single center, randomized controlled trial	Lianquan (CV 23), Shanglianquan (depression between the hyoid bone and the lower border of the mandible, Extra), and Yifeng (TE 17), Fengchi (GB 20), Wangu (GB 12), Fengfu (GV 16), Yamen (GV 15), and Neidaying (depression of 1 inch below the anterior margin of the mandible, Extra).	Penetration-Aspiration Scale (PAS)	PAS for paste, SMD 2.06, 95% CI [1.60, 2.53] PAS for liquid, SMD -0.02, 95% CI [-0.39, 0.35]	No participants withdrew from the study because of an AE.
Fukuda et al. (2016)	13	A prospective case series study.	ST36, SP6 and LR3 in the legs; LI4, LI11 in the arms; GB20 in the neck; and BL18, BL23 in the back(all bilateral)	pressure with which the tongue grinds food against the frontal palate in the oral cavity	SMD 0.46, 95% CI [-0.32, 1.24]	No adverse effects related to the acupuncture.

### 4.2 Acupuncture may exert an ameliorative effect on Parkinson’s symptoms by regulating the gut-brain axis

On the one hand, acupuncture can improve the imbalance of intestinal flora. Through 16S rRNA sequence analysis, Jang JH et al. observed that acupuncture changed the relative abundance of 18 genera in PD mice, of which Butyricimonas, Holdemania, Frisingicoccus, Gracilibacter, Phocea, and Aestuariispira showed significant correlations with the improvement of various dysfunctions and anxiety (Jang et al., 2020). Han et al. (2021) found out that electroacupuncture may alleviate behavioral deficits by modulating gut microbiota and suppress inflammation in a mouse model of PD. Research has shown that acupuncture may increase the abundance levels of Roseburia (Feng et al., 2025), Faecalibacterium (Bao et al., 2022), and Prevotellaceae (Wang H. et al., 2023).

On the other hand, acupuncture can also regulate the expression of *α*-syn. Firstly, acupuncture blocks intestinal inflammation by inhibiting NLRP3 inflammasome activation (Guo et al., 2024) and reduces α-syn expression through anti-inflammatory and antioxidant activities (Deng et al., 2015). Secondly, acupuncture can also promote the autophagic clearance of α-syn (Zhang et al., 2024). Furthermore, acupuncture can directly reduce the expression of α-syn in the SN (Yeo et al., 2020). Finally, acupuncture can inhibit the apoptosis pathway, upregulate endogenous brain-derived neurotrophic factor (BDNF), and activate downstream PI3k/AKT to degrade α-syn (Lin et al., 2017). In an MTPT-induced mouse model of PD, acupuncture stimulation of GB34 and LR3 attenuated not only the decrease of tyrosine hydroxylase in the SN but also the elevation of SN α-syn (Yeo et al., 2020). In a mouse model of rotenone-induced PD, electroacupuncture of ST25 (Tianshu), ST37 (Shangjuxu), LI11 (Quchi), and DU24 (Shenting) acupoints reduces the expression of alpha-syn in the colon and substantia nigra, thereby delaying the onset time of behavioral disorders in mice (Ma et al., 2021).

Acupuncture also exerts neuroprotective effects on PD. Firstly, acupuncture can exert neuroprotective effects by increasing L-dopa and 5-HT levels (Nguyen et al., 2025). Secondly, acupuncture also up-regulates α7-nicotinic acetylcholine receptors (α7nAChR) through central cholinergic mechanisms, thus promoting the release of glial cell line-derived neurotrophic factor (GDNF) by EGCs, Ultimately, intestinal neurons are protected (Zhang et al., 2024). Finally, acupuncture can also directly upregulate GDNF and BDNF in the SN and striatum (Pak et al., 2020), thereby exerting a protective effect on dopaminergic neurons. A recent meta-analysis pointed out the protective effects of acupuncture on dopaminergic neurons in a rodent model of PD (Ko et al., 2019). Earlier experiments have also illustrated that acupuncture prevents 6-hydroxydopamine-induced neuronal death in the nigrostriatal dopaminergic system in a rat model of PD (Park et al., 2003).

In addition, acupuncture can improve neuroinflammation. Firstly, acupuncture can reduce various pro-inflammatory factors such as TNF-α, IL-1β, and IL-6 while increasing the anti-inflammatory factor IL-10 (Jeon et al., 2025). Secondly, acupuncture regulates the pathways related to inflammatory responses. Experiments conducted Jeon et al. have shown that acupuncture can inhibit the MAPK signaling pathway (Jeon et al., 2025), thereby improving neuroinflammation. Finally, acupuncture can regulate the function of immune cells such as T lymphocytes (Kim et al., 2024). Tsai et al. found that acupuncture of Baihui and Si Shencong improved neurological function and reduced serum inflammation in ischemic stroke patients (Tsai et al., 2024). A recent meta-analysis suggested that acupuncture reduced inflammation by modulating cytokines (Lee and Kim, 2022).

Finally, acupuncture can inhibit astrocyte proliferation and microglia polarization (Li et al., 2025). Earlier experiments have demonstrated that acupuncture of GB34 and LR3 inhibits MPTP-induced microglia activation and inflammatory responses in a PD model (Kang et al., 2007). Recently, Zou et al. have indicated that electroacupuncture also inhibits microglial cell M1 polarization, which in turn alleviates PD symptoms in the MTPT-induced mouse model of PD (Zou et al., 2024).

**Figure 1:**
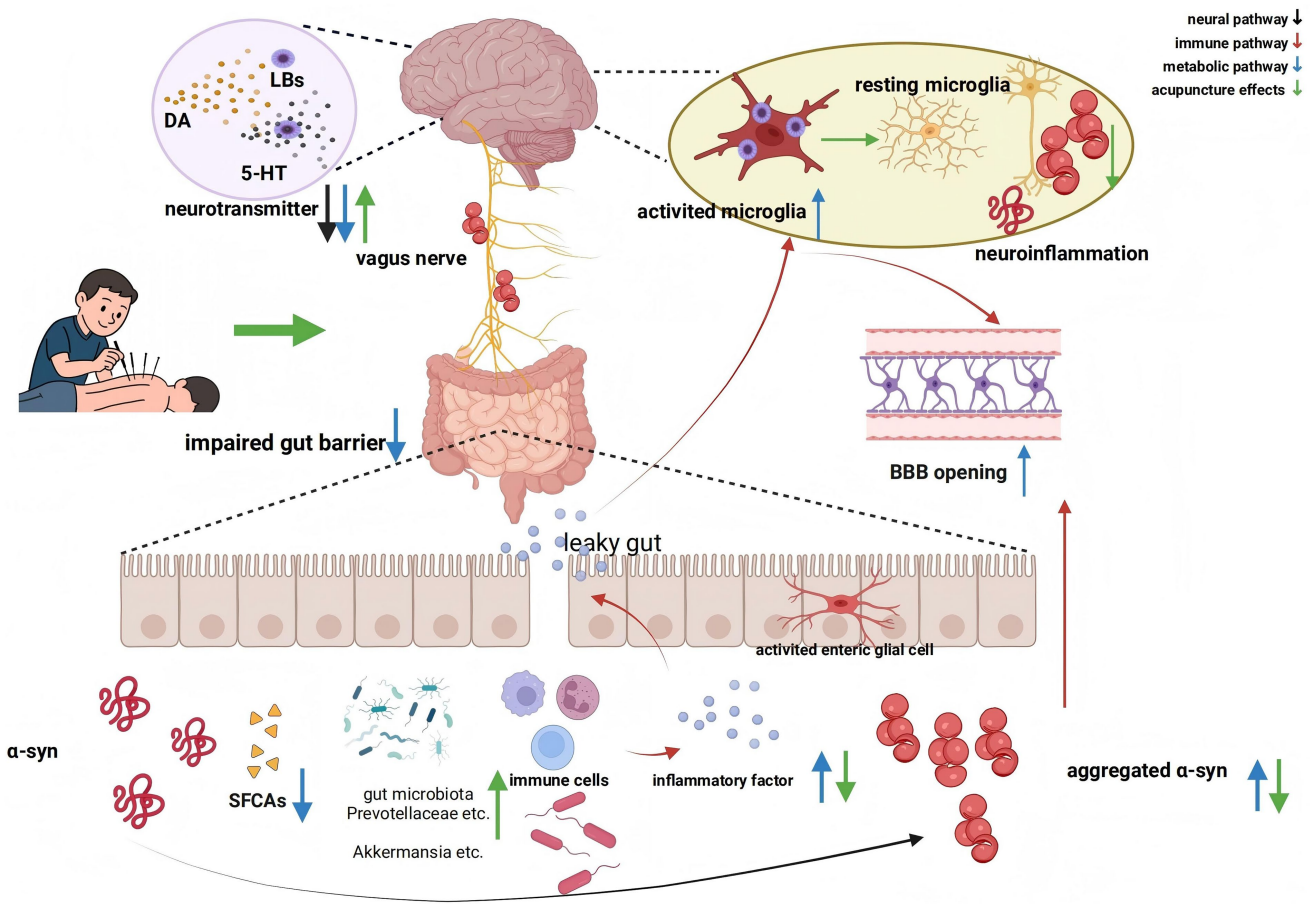
Acupuncture regulates Parkinson’s disease through the gut-brain axis. Due to changes in the intestinal microbiota, and bacterial infections, leading to the aggregated α-syn. Then they are transmitted to the CNS via the vagus nerve and form neurotoxic LBs in the brain, which lead to PD. EGCs activation impairs the gut barrier and increases peripheral proinflammatory factors, activates microglia, opens the BBB, and then allows aggregated α-syn to enter the brain. Changes in the gut microbiota may also lead to a reduction in SCFAs. Acupuncture mainly affects the gut-brain axis by improving intestinal flora dysbiosis, interfering with α-syn expression, protecting neurological function, anti-inflammation, and affecting glial cells. This figure was created by BioRender (www.biorender.com).

In conclusion, acupuncture mainly affects the GBA by improving intestinal flora dysbiosis, interfering with α-syn expression, protecting neurological function, reducing inflammation, and affecting glial cells, which in turn improves PD (Xu et al., 2025) (Figure 1).

## 5 Conclusion and future direction

In summary, PD is a complex neurodegenerative disease that involves dysregulation of multiple systems, including the nervous system, endocrine system, and immune system. In recent years, GBA has emerged as a new area of research, providing new insights into the pathogenesis of PD. The preliminary evidence has suggested that acupuncture, as a traditional Chinese medicine therapy, has potential in treating dyskinesia and non-motor symptoms of PD. It represents a promising clinical tool for the treatment of PD, although the mechanism is still unclear. Acupuncture may treat PD not only by modulating the gut microbiota, neurotransmitters and the autonomic nervous system but also by affecting glial cells acting on the GBA. However, there are still many limitations and challenges at this stage. Current meta-analyses have also pointed out that most studies face a high risk of bias of blinding and regional differences. It is recommended that high-quality, multi-center RCTs be conducted in multiple regions around the world. In addition, acupuncture treatment often relies on practitioners’ clinical experience and lacks experimental data to validate its scientific basis and feasibility, which makes it is necessary to establish standardized acupuncture protocols as soon as possible. Despite the demonstrated clinical efficacy of acupuncture in the treatment of PD, there remains very limited research on the mechanism of acupuncture in treating PD through GBA. In future studies, basic science with clinical practice should be further combined to explore the specific regulatory mechanisms of GBA in PD, contributing feasible and effective methods to the treatment of PD.

## References

[ref1] Abdelnaby R. Elsayed M. Mohamed K. A. Dardeer K. T. Sonbol Y. T. Elgenidy A.. (2021). Vagus nerve ultrasonography in Parkinson’s disease: a systematic review and meta-analysis. Auton. Neurosci. 234:102835. doi: 10.1016/j.autneu.2021.102835, PMID: 34166995

[ref2] Al-Bachari S. Naish J. H. Parker G. J. M. Emsley H. C. A. Parkes L. M. (2020). Blood-brain barrier leakage is increased in Parkinson’s disease. Front. Physiol. 11:593026. doi: 10.3389/fphys.2020.593026, PMID: 33414722 PMC7784911

[ref3] Antonelli F. Strafella A. P. (2014). Behavioral disorders in Parkinson’s disease: the role of dopamine. Park. Relat. Disord. 20, S10–S12. doi: 10.1016/S1353-8020(13)70005-1 24262157

[ref4] Baert F. Matthys C. Maselyne J. Van Poucke C. Van Coillie E. Bergmans B.. (2021). Parkinson’s disease patients’ short chain fatty acids production capacity after in vitro fecal fiber fermentation. NPJ Parkinson’s Dis. 7:72. doi: 10.1038/s41531-021-00215-5, PMID: 34389734 PMC8363715

[ref5] Bai F. You L. Lei H. Li X. (2024). Association between increased and decreased gut microbiota abundance and Parkinson’s disease: a systematic review and subgroup meta-analysis. Exp. Gerontol. 191:112444. doi: 10.1016/j.exger.2024.112444, PMID: 38679353

[ref6] Bao C. Wu L. Wang D. Chen L. Jin X. Shi Y.. (2022). Acupuncture improves the symptoms, intestinal microbiota, and inflammation of patients with mild to moderate crohn’s disease: a randomized controlled trial. Eclinicalmedicine 45:101300. doi: 10.1016/j.eclinm.2022.101300, PMID: 35198926 PMC8850329

[ref7] Borghammer P. Hamani C. (2017). Preventing Parkinson disease by vagotomy: fact or fiction? Neurology 88, 1982–1983. doi: 10.1212/WNL.0000000000003969, PMID: 28446643

[ref8] Bosch-Barceló P. Martínez-Navarro O. Masbernat-Almenara M. Tersa-Miralles C. Pakarinen A. Fernández-Lago H. (2025). Gamification integration in technological devices for motor rehabilitation in Parkinson disease: scoping review. JMIR Serious Games 13:e69433. doi: 10.2196/69433, PMID: 40614259 PMC12252148

[ref9] Bostick J. W. Schonhoff A. M. Mazmanian S. K. (2022). Gut microbiome-mediated regulation of neuroinflammation. Curr. Opin. Immunol. 76:102177. doi: 10.1016/j.coi.2022.102177, PMID: 35462279 PMC9167715

[ref10] Braak H. de Vos R. A. I. Bohl J. Del Tredici K. (2006). Gastric alpha-synuclein immunoreactive inclusions in meissner’s and Auerbach’s plexuses in cases staged for Parkinson’s disease-related brain pathology. Neurosci. Lett. 396, 67–72. doi: 10.1016/j.neulet.2005.11.012, PMID: 16330147

[ref11] Braak H. Del Tredici K. Rüb U. de Vos R. A. I. Jansen Steur E. N. H. Braak E. (2003). Staging of brain pathology related to sporadic Parkinson’s disease. Neurobiol. Aging 24, 197–211. doi: 10.1016/s0197-4580(02)00065-9, PMID: 12498954

[ref12] Bu L.-L. Huang K.-X. Zheng D.-Z. Lin D.-Y. Chen Y. Jing X.-N.. (2020). Alpha-synuclein accumulation and its phosphorylation in the enteric nervous system of patients without neurodegeneration: an explorative study. Front. Aging Neurosci. 12:575481. doi: 10.3389/fnagi.2020.575481, PMID: 33328957 PMC7719782

[ref13] Cao L. Li X. Li M. Yao L. Hou L. Zhang W.. (2020). The effectiveness of acupuncture for Parkinson’s disease: an overview of systematic reviews. Complement. Ther. Med. 50:102383. doi: 10.1016/j.ctim.2020.102383, PMID: 32444048

[ref14] Cheng Y. Tan G. Zhu Q. Wang C. Ruan G. Ying S.. (2023). Efficacy of fecal microbiota transplantation in patients with Parkinson’s disease: clinical trial results from a randomized, placebo-controlled design. Gut Microbes 15:2284247. doi: 10.1080/19490976.2023.2284247, PMID: 38057970 PMC10841011

[ref15] Chenghan M. Wanxin L. Bangcheng Z. Yao H. Qinxi L. Ting Z.. (2025). Short-chain fatty acids mediate gut microbiota-brain communication and protect the blood-brain barrier integrity. Ann. N. Y. Acad. Sci. 1545, 116–131. doi: 10.1111/nyas.15299, PMID: 39998158

[ref16] Cho S.-Y. Lee Y.-E. Doo K.-H. Lee J.-H. Jung W.-S. Moon S.-K.. (2018). Efficacy of combined treatment with acupuncture and bee venom acupuncture as an adjunctive treatment for Parkinson’s disease. J. Altern. Complement. Med. 24, 25–32. doi: 10.1089/acm.2016.0250, PMID: 28753030

[ref17] Chu C. Yu L. Li Y. Guo G. Zhai Q. Chen W.. (2023). Meta-analysis of randomized controlled trials of the effects of probiotics in Parkinson’s disease. Food Funct. 14, 3406–3422. doi: 10.1039/d2fo03825k, PMID: 36974511

[ref18] Claudino Dos Santos J. C. Oliveira L. F. Noleto F. M. CTP G. GAC B. GSB V. (2023). Role of enteric glia and microbiota-gut-brain axis in Parkinson disease pathogenesis. Ageing Res. Rev. 84:101812. doi: 10.1016/j.arr.2022.101812, PMID: 36455790

[ref19] Collins S. M. Surette M. Bercik P. (2012). The interplay between the intestinal microbiota and the brain. Nat. Rev. Microbiol. 10, 735–742. doi: 10.1038/nrmicro2876, PMID: 23000955

[ref20] Cox M. J. Cookson W. O. C. M. Moffatt M. F. (2013). Sequencing the human microbiome in health and disease. Hum. Mol. Genet. 22, R88–R94. doi: 10.1093/hmg/ddt398, PMID: 23943792

[ref21] Cui C. Han Y. Li H. Yu H. Zhang B. Li G. (2022). Curcumin-driven reprogramming of the gut microbiota and metabolome ameliorates motor deficits and neuroinflammation in a mouse model of Parkinson’s disease. Front. Cell. Infect. Microbiol. 12:887407. doi: 10.3389/fcimb.2022.887407, PMID: 36034698 PMC9400544

[ref22] Cushing K. Alvarado D. M. Ciorba M. A. (2015). Butyrate and mucosal inflammation: new scientific evidence supports clinical observation. Clin. Transl. Gastroenterol. 6:e108. doi: 10.1038/ctg.2015.34, PMID: 26312412 PMC4816278

[ref23] Del Tredici K. Braak H. (2016). Review: sporadic Parkinson’s disease: development and distribution of α-synuclein pathology. Neuropathol. Appl. Neurobiol. 42, 33–50. doi: 10.1111/nan.12298, PMID: 26662475

[ref24] Deng J. Lv E. Yang J. Gong X. Zhang W. Liang X.. (2015). Electroacupuncture remediates glial dysfunction and ameliorates neurodegeneration in the astrocytic α-synuclein mutant mouse model. J. Neuroinflammation 12:103. doi: 10.1186/s12974-015-0302-z, PMID: 26016857 PMC4449593

[ref25] Di Vincenzo F. Del Gaudio A. Petito V. Lopetuso L. R. Scaldaferri F. (2024). Gut microbiota, intestinal permeability, and systemic inflammation: a narrative review. Intern. Emerg. Med. 19, 275–293. doi: 10.1007/s11739-023-03374-w, PMID: 37505311 PMC10954893

[ref26] Dicks L. M. T. (2023). Our mental health is determined by an intrinsic interplay between the central nervous system, enteric nerves, and gut microbiota. Int. J. Mol. Sci. 25:38. doi: 10.3390/ijms25010038, PMID: 38203207 PMC10778721

[ref27] Dorsey E. R. Constantinescu R. Thompson J. P. Biglan K. M. Holloway R. G. Kieburtz K.. (2007). Projected number of people with Parkinson disease in the most populous nations, 2005 through 2030. Neurology 68, 384–386. doi: 10.1212/01.wnl.0000247740.47667.03, PMID: 17082464

[ref28] Dowling L. R. Strazzari M. R. Keely S. Kaiko G. E. (2022). Enteric nervous system and intestinal epithelial regulation of the gut-brain axis. J. Allergy Clin. Immunol. 150, 513–522. doi: 10.1016/j.jaci.2022.07.015, PMID: 36075637

[ref29] Emmi A. Sandre M. Russo F. P. Tombesi G. Garrì F. Campagnolo M.. (2023). Duodenal alpha-synuclein pathology and enteric gliosis in advanced Parkinson’s disease. Mov. Disord. 38, 885–894. doi: 10.1002/mds.29358, PMID: 36847308

[ref30] Erny D. Hrabě de Angelis A. L. Jaitin D. Wieghofer P. Staszewski O. David E.. (2015). Host microbiota constantly control maturation and function of microglia in the CNS. Nat. Neurosci. 18, 965–977. doi: 10.1038/nn.4030, PMID: 26030851 PMC5528863

[ref31] Fan J.-Q. Lu W.-J. Tan W.-Q. Liu X. Wang Y.-T. Wang N.-B.. (2022). Effectiveness of acupuncture for anxiety among patients with Parkinson disease: a randomized clinical trial. JAMA Netw. Open 5:e2232133. doi: 10.1001/jamanetworkopen.2022.32133, PMID: 36129711 PMC9494193

[ref32] Fang X. Liu S. Muhammad B. Zheng M. Ge X. Xu Y.. (2024). Gut microbiota dysbiosis contributes to α-synuclein-related pathology associated with C/EBPβ/AEP signaling activation in a mouse model of Parkinson’s disease. Neural Regen. Res. 19, 2081–2088. doi: 10.4103/1673-5374.391191, PMID: 38227539 PMC11040317

[ref33] Feng C. Pan H. Zhang Y. Ye Z. Zhou Y. Zou H.. (2025). Electroacupuncture alleviates neuropathic pain and negative emotion in mice by regulating gut microbiota. J. Pain Res. 18, 341–352. doi: 10.2147/JPR.S501642, PMID: 39867538 PMC11761536

[ref34] Fernandez C. G. Hamby M. E. McReynolds M. L. Ray W. J. (2019). The role of APOE4 in disrupting the homeostatic functions of astrocytes and microglia in aging and Alzheimer’s disease. Front. Aging Neurosci. 11:14. doi: 10.3389/fnagi.2019.00014, PMID: 30804776 PMC6378415

[ref35] Forno L. S. (1987). The lewy body in Parkinson’s disease. Adv. Neurol. 45, 35–43, PMID: 3030070

[ref36] Forsyth C. B. Shannon K. M. Kordower J. H. Voigt R. M. Shaikh M. Jaglin J. A.. (2011). Increased intestinal permeability correlates with sigmoid mucosa alpha-synuclein staining and endotoxin exposure markers in early Parkinson’s disease. PLoS One 6:e28032. doi: 10.1371/journal.pone.0028032, PMID: 22145021 PMC3228722

[ref37] Fukuda S. Kuriyama N. Tsuru H. Egawa M. (2016). Immediate effects of acupuncture on tongue pressure including swallowing reflex latency in Parkinson’s disease. J. Br. Med. Acupunct. Soc. 34, 59–61. doi: 10.1136/acupmed-2015-010811, PMID: 26296358

[ref38] Fülling C. Dinan T. G. Cryan J. F. (2019). Gut microbe to brain signaling: what happens in vagus …. Neuron 101, 998–1002. doi: 10.1016/j.neuron.2019.02.008, PMID: 30897366

[ref39] Gao K. Mu C.-L. Farzi A. Zhu W.-Y. (2020). Tryptophan metabolism: a link between the gut microbiota and brain. Adv. Nutr. 11, 709–723. doi: 10.1093/advances/nmz127, PMID: 31825083 PMC7231603

[ref40] Goya M. E. Xue F. Sampedro-Torres-Quevedo C. Arnaouteli S. Riquelme-Dominguez L. Romanowski A.. (2020). Probiotic *bacillus subtilis* protects against α-synuclein aggregation in *C. elegans*. Cell Rep. 30, 367–380.e7. doi: 10.1016/j.celrep.2019.12.078 31940482 PMC6963774

[ref41] Gray M. T. Woulfe J. M. (2015). Striatal blood-brain barrier permeability in Parkinson’s disease. J. Int. Soc. Cereb. Blood Flow Metab. 35, 747–750. doi: 10.1038/jcbfm.2015.32, PMID: 25757748 PMC4420870

[ref42] Guo L. Hu H. Jiang N. Yang H. Sun X. Xia H.. (2024). Electroacupuncture blocked motor dysfunction and gut barrier damage by modulating intestinal NLRP3 inflammasome in MPTP-induced Parkinson’s disease mice. Heliyon 10:e30819. doi: 10.1016/j.heliyon.2024.e30819, PMID: 38774094 PMC11107113

[ref43] Han Q.-Q. Fu Y. Le J.-M. Pilot A. Cheng S. Chen P.-Q.. (2021). Electroacupuncture may alleviate behavioral defects via modulation of gut microbiota in a mouse model of Parkinson’s disease. Acupunct. Med; J. Br. Med. Acupunct. Soc. 39, 501–511. doi: 10.1177/0964528421990658, PMID: 33557583

[ref44] Han Y. Wang B. Gao H. He C. Hua R. Liang C.. (2022). Vagus nerve and underlying impact on the gut microbiota-brain axis in behavior and neurodegenerative diseases. J. Inflamm. Res. 15, 6213–6230. doi: 10.2147/JIR.S384949, PMID: 36386584 PMC9656367

[ref45] Hauser R. A. (2009). Levodopa: past, present, and future. Eur. Neurol. 62, 1–8. doi: 10.1159/000215875, PMID: 19407449

[ref46] Hirayama M. Ohno K. (2021). Parkinson’s disease and gut microbiota. Ann. Nutr. Metab. 77, 28–35. doi: 10.1159/000518147, PMID: 34500451

[ref47] Holmqvist S. Chutna O. Bousset L. Aldrin-Kirk P. Li W. Björklund T.. (2014). Direct evidence of Parkinson pathology spread from the gastrointestinal tract to the brain in rats. Acta Neuropathol. 128, 805–820. doi: 10.1007/s00401-014-1343-6, PMID: 25296989

[ref48] Hong C.-T. Chen J.-H. Huang T.-W. (2022). Probiotics treatment for Parkinson disease: a systematic review and meta-analysis of clinical trials. Aging 14, 7014–7025. doi: 10.18632/aging.204266, PMID: 36084951 PMC9512504

[ref49] Hou Y. Li X. Liu C. Zhang M. Zhang X. Ge S.. (2021a). Neuroprotective effects of short-chain fatty acids in MPTP induced mice model of Parkinson’s disease. Exp. Gerontol. 150:111376. doi: 10.1016/j.exger.2021.111376, PMID: 33905875

[ref50] Hou Y. Ning B. Liu Y. Liu Y. Fu W. Wen Z. (2021b). Effectiveness and safety of moxibustion for Parkinson disease: a protocol for systematic review and meta-analysis. Medicine (Baltimore) 100:e26256. doi: 10.1097/MD.0000000000026256, PMID: 34115018 PMC8202601

[ref51] Huang T. Shi H. Xu Y. Ji L. (2021). The gut microbiota metabolite propionate ameliorates intestinal epithelial barrier dysfunction-mediated Parkinson’s disease via the AKT signaling pathway. Neuroreport 32, 244–251. doi: 10.1097/WNR.0000000000001585 33470765

[ref52] Jacobs B. L. Azmitia E. C. (1992). Structure and function of the brain serotonin system. Physiol. Rev. 72, 165–229. doi: 10.1152/physrev.1992.72.1.165, PMID: 1731370

[ref53] Jameson K. G. Hsiao E. Y. (2019). A novel pathway for microbial metabolism of levodopa. Nat. Med. 25, 1195–1197. doi: 10.1038/s41591-019-0544-x, PMID: 31388180 PMC7004239

[ref54] Jang J.-H. Yeom M.-J. Ahn S. Oh J.-Y. Ji S. Kim T.-H.. (2020). Acupuncture inhibits neuroinflammation and gut microbial dysbiosis in a mouse model of Parkinson’s disease. Brain Behav. Immun. 89, 641–655. doi: 10.1016/j.bbi.2020.08.015, PMID: 32827699

[ref55] Jellinger K. A. (2015). Neuropathobiology of non-motor symptoms in parkinson disease. J. Neural Transm. 122, 1429–1440. doi: 10.1007/s00702-015-1405-5 25976432

[ref56] Jeon H. Oh J.-Y. Ahn S. Yeom M. Ha I. J. Son H.-S.. (2025). Invasive laser acupuncture targeting muscle: a novel approach to protect dopaminergic neurons and reduce neuroinflammation in a brain of Parkinson’s disease model. Chin. Med. 20:59. doi: 10.1186/s13020-025-01104-2, PMID: 40336061 PMC12057028

[ref57] Jiang F. Yang T. Yin H. Guo Y. Namba H. Sun Z.. (2018). Evidence for the use of acupuncture in treating Parkinson’s disease: update of information from the past 5 years, a mini review of the literature. Front. Neurol. 9:596. doi: 10.3389/fneur.2018.00596, PMID: 30090084 PMC6068266

[ref58] Kabouridis P. S. Lasrado R. McCallum S. Chng S. H. Snippert H. J. Clevers H.. (2015). Microbiota controls the homeostasis of glial cells in the gut lamina propria. Neuron 85, 289–295. doi: 10.1016/j.neuron.2014.12.037, PMID: 25578362 PMC4306542

[ref59] Kalia L. V. Lang A. E. (2015). Parkinson’s disease. Lancet 386, 896–912. doi: 10.1016/S0140-6736(14)61393-3, PMID: 25904081

[ref60] Kalyanaraman B. Cheng C. Hardy M. (2024). Gut microbiome, short-chain fatty acids, alpha-synuclein, neuroinflammation, and ROS/RNS: relevance to Parkinson’s disease and therapeutic implications. Redox Biol. 71:103092. doi: 10.1016/j.redox.2024.103092 38377788 PMC10891329

[ref61] Kang J. M. Park P. J. Choi Y. G. Choe I. H. Park J. H. Kim Y. S.. (2007). Acupuncture inhibits microglial activation and inflammatory events in the MPTP-induced mouse model. Brain Res. 1131, 211–219. doi: 10.1016/j.brainres.2006.10.089, PMID: 17173870

[ref62] Kelly C. J. Zheng L. Campbell C. Saeedi B. Scholz C. C. Bayless A. J.. (2015). Crosstalk between microbiota-derived short-chain fatty acids and intestinal epithelial HIF augments tissue barrier function. Cell Host Microbe 17, 662–671. doi: 10.1016/j.chom.2015.03.005, PMID: 25865369 PMC4433427

[ref63] Kim J. H. Choi Y. Kim J. S. Lee H. Ju I. G. Yoo N. Y.. (2024). Stimulation of microneedles alleviates pathology of Parkinson’s disease in mice by regulating the CD4+/CD8+ cells from the periphery to the brain. Front. Immunol. 15:1454102. doi: 10.3389/fimmu.2024.1454102, PMID: 39628485 PMC11611716

[ref64] Kin I. Sasaki T. Yasuhara T. Kameda M. Agari T. Okazaki M.. (2021). Vagus nerve stimulation with mild stimulation intensity exerts anti-inflammatory and neuroprotective effects in Parkinson’s disease model rats. Biomedicines 9:789. doi: 10.3390/biomedicines9070789, PMID: 34356853 PMC8301489

[ref65] Kleine Bardenhorst S. Cereda E. Severgnini M. Barichella M. Pezzoli G. Keshavarzian A.. (2023). Gut microbiota dysbiosis in Parkinson disease: a systematic review and pooled analysis. Eur. J. Neurol. 30, 3581–3594. doi: 10.1111/ene.15671, PMID: 36593694

[ref66] Ko J. H. Lee H. Kim S.-N. Park H.-J. (2019). Does acupuncture protect dopamine neurons in Parkinson’s disease rodent model?: a systematic review and meta-analysis. Front. Aging Neurosci. 11:102. doi: 10.3389/fnagi.2019.00102, PMID: 31139074 PMC6517785

[ref67] Korsten S. G. P. J. Vromans H. Garssen J. Willemsen L. E. M. (2023). Butyrate protects barrier integrity and suppresses immune activation in a caco-2/PBMC co-culture model while HDAC inhibition mimics butyrate in restoring cytokine-induced barrier disruption. Nutrients 15:2760. doi: 10.3390/nu15122760, PMID: 37375664 PMC10305054

[ref68] Koutzoumis D. N. Vergara M. Pino J. Buddendorff J. Khoshbouei H. Mandel R. J.. (2020). Alterations of the gut microbiota with antibiotics protects dopamine neuron loss and improve motor deficits in a pharmacological rodent model of Parkinson’s disease. Exp. Neurol. 325:113159. doi: 10.1016/j.expneurol.2019.113159, PMID: 31843492

[ref69] La Vitola P. Balducci C. Baroni M. Artioli L. Santamaria G. Castiglioni M.. (2021). Peripheral inflammation exacerbates α-synuclein toxicity and neuropathology in Parkinson’s models. Neuropathol. Appl. Neurobiol. 47, 43–60. doi: 10.1111/nan.12644, PMID: 32696999

[ref70] Lan G. Wang P. Chan R. B. Liu Z. Yu Z. Liu X.. (2022). Astrocytic VEGFA: an essential mediator in blood-brain-barrier disruption in Parkinson’s disease. Glia 70, 337–353. doi: 10.1002/glia.24109, PMID: 34713920

[ref71] Lashuel H. A. Overk C. R. Oueslati A. Masliah E. (2013). The many faces of α-synuclein: from structure and toxicity to therapeutic target. Nat. Rev. Neurosci. 14, 38–48. doi: 10.1038/nrn3406, PMID: 23254192 PMC4295774

[ref72] Lau K. Kotzur R. Richter F. (2024). Blood-brain barrier alterations and their impact on Parkinson’s disease pathogenesis and therapy. Transl. Neurodegener. 13:37. doi: 10.1186/s40035-024-00430-z, PMID: 39075566 PMC11285262

[ref73] Le Floc’h N. Otten W. Merlot E. (2011). Tryptophan metabolism, from nutrition to potential therapeutic applications. Amino Acids 41, 1195–1205. doi: 10.1007/s00726-010-0752-7, PMID: 20872026

[ref74] Lee S. Kim S. N. (2022). The effect of acupuncture on modulating inflammatory cytokines in rodent animal models of respiratory disease: a systematic review and meta-analysis. Front. Immunol. 13:878463. doi: 10.3389/fimmu.2022.878463, PMID: 35784312 PMC9241441

[ref75] Lei S. Fan J. Liu X. Xv X. Zhang J. Zhou Z.. (2023). Qualitative and quantitative meta-analysis of acupuncture effects on the motor function of Parkinson’s disease patients. Front. Neurosci. 17:1125626. doi: 10.3389/fnins.2023.1125626, PMID: 37229426 PMC10203172

[ref76] Lei Q. Wu T. Wu J. Hu X. Guan Y. Wang Y.. (2021). Roles of α-synuclein in gastrointestinal microbiome dysbiosis-related Parkinson’s disease progression (review). Mol. Med. Rep. 24:734. doi: 10.3892/mmr.2021.12374, PMID: 34414447 PMC8404091

[ref77] Li J. Guo Y. Zeng X. Tian H. Han Y. Cai S.. (2025). Electroacupuncture stimulation inhibited astrogliosis and microglia polarisation to alleviate spinal cord injury via janus kinase 2/signal transducer and activator of transcription 3 signalling pathway. Folia Histochem. Cytobiol. 63, 28–40. doi: 10.5603/fhc.104273, PMID: 40421823

[ref78] Li Y. J. Leong I.-I. Fan J.-Q. Yan M.-Y. Liu X. Lu W.-J.. (2023). Efficacy of acupuncture for the treatment of Parkinson’s disease-related constipation (PDC): a randomized controlled trial. Front. Neurosci. 17:1126080. doi: 10.3389/fnins.2023.1126080, PMID: 36866329 PMC9972583

[ref79] Li K. Xu S. Wang R. Zou X. Liu H. Fan C.. (2023). Electroacupuncture for motor dysfunction and constipation in patients with Parkinson’s disease: a randomised controlled multi-Centre trial. Eclinicalmedicine 56:101814. doi: 10.1016/j.eclinm.2022.101814, PMID: 36691434 PMC9860357

[ref80] Lin J.-G. Chen C.-J. Yang H.-B. Chen Y.-H. Hung S.-Y. (2017). Electroacupuncture promotes recovery of motor function and reduces dopaminergic neuron degeneration in rodent models of Parkinson’s disease. Int. J. Mol. Sci. 18:1846. doi: 10.3390/ijms18091846, PMID: 28837077 PMC5618495

[ref81] Lipski J. Nistico R. Berretta N. Guatteo E. Bernardi G. Mercuri N. B. (2011). L-DOPA: a scapegoat for accelerated neurodegeneration in Parkinson’s disease? Prog. Neurobiol. 94, 389–407. doi: 10.1016/j.pneurobio.2011.06.005, PMID: 21723913

[ref82] Ma X. Qiao H.-F. Wang Q. Wang Y. Yuan W. Liu Z.-B. (2021). Effect of electroacupuncture on expression of α-syn in colon and substantia nigra of Parkinson’s disease mice. Zhen Ci Yan Ji 46, 362–367. doi: 10.13702/j.1000-0607.200672, PMID: 34085457

[ref83] Mahbub N. U. Islam M. M. Hong S.-T. Chung H.-J. (2024). Dysbiosis of the gut microbiota and its effect on α-synuclein and prion protein misfolding: consequences for neurodegeneration. Front. Cell. Infect. Microbiol. 14:1348279. doi: 10.3389/fcimb.2024.1348279, PMID: 38435303 PMC10904658

[ref84] Mawe G. M. Hoffman J. M. (2013). Serotonin signalling in the gut--functions, dysfunctions and therapeutic targets. Nat. Rev. Gastroenterol. Hepatol. 10, 473–486. doi: 10.1038/nrgastro.2013.105, PMID: 23797870 PMC4048923

[ref85] Mi W. Meng M. Xu F. Sun L. (2024). Efficacy of acupuncture as adjunct therapy for sleep disorders in Parkinson’s disease: a systematic review and meta-analysis. Complement. Ther. Med. 82:103044. doi: 10.1016/j.ctim.2024.103044, PMID: 38679147

[ref86] Mirzaei R. Bouzari B. Hosseini-Fard S. R. Mazaheri M. Ahmadyousefi Y. Abdi M.. (2021). Role of microbiota-derived short-chain fatty acids in nervous system disorders. Biomed. Pharmacother. 139:111661. doi: 10.1016/j.biopha.2021.111661, PMID: 34243604

[ref87] Montalbán-Rodríguez A. Abalo R. López-Gómez L. (2024). From the gut to the brain: the role of enteric glial cells and their involvement in the pathogenesis of Parkinson’s disease. Int. J. Mol. Sci. 25:1294. doi: 10.3390/ijms25021294, PMID: 38279293 PMC10816228

[ref88] Mulak A. Bonaz B. (2015). Brain-gut-microbiota axis in Parkinson’s disease. World J. Gastroenterol. 21, 10609–10620. doi: 10.3748/wjg.v21.i37.10609, PMID: 26457021 PMC4588083

[ref89] Natale G. Ryskalin L. Morucci G. Lazzeri G. Frati A. Fornai F. (2021). The baseline structure of the enteric nervous system and its role in Parkinson’s disease. Life 11:732. doi: 10.3390/life11080732, PMID: 34440476 PMC8400095

[ref90] Nguyen H. T.-M. Lee D.-Y. Hsieh C.-L. (2025). Auricular acupuncture plays a neuroprotective role in 6-hydroxydopamine-induced Parkinson’s disease in rats. J. Tradit. Complement. Med. 15, 128–139. doi: 10.1016/j.jtcme.2024.05.008, PMID: 40060148 PMC11883624

[ref91] Nie S. Wang J. Deng Y. Ye Z. Ge Y. (2022). Inflammatory microbes and genes as potential biomarkers of Parkinson’s disease. Npj Biofilms Microbiomes 8:101. doi: 10.1038/s41522-022-00367-z, PMID: 36564391 PMC9789082

[ref92] Noh H. Kwon S. Cho S.-Y. Jung W.-S. Moon S.-K. Park J.-M.. (2017). Effectiveness and safety of acupuncture in the treatment of Parkinson’s disease: a systematic review and meta-analysis of randomized controlled trials. Complement. Ther. Med. 34, 86–103. doi: 10.1016/j.ctim.2017.08.005, PMID: 28917379

[ref93] Paiva I. Pinho R. Pavlou M. A. Hennion M. Wales P. Schütz A.-L.. (2017). Sodium butyrate rescues dopaminergic cells from alpha-synuclein-induced transcriptional deregulation and DNA damage. Hum. Mol. Genet. 26, 2231–2246. doi: 10.1093/hmg/ddx114, PMID: 28369321

[ref94] Pak M. E. Ahn S. M. Jung D. H. Lee H. J. Ha K. T. Shin H. K.. (2020). Electroacupuncture therapy ameliorates motor dysfunction via brain-derived neurotrophic factor and glial cell line-derived neurotrophic factor in a mouse model of Parkinson’s disease. J. Gerontol. A Biol. Sci. Med. Sci. 75, 712–721. doi: 10.1093/gerona/glz256, PMID: 31644786

[ref95] Park H. J. Lim S. Joo W. S. Yin C. S. Lee H. S. Lee H. J.. (2003). Acupuncture prevents 6-hydroxydopamine-induced neuronal death in the nigrostriatal dopaminergic system in the rat Parkinson’s disease model. Exp. Neurol. 180, 93–98. doi: 10.1016/s0014-4886(02)00031-6, PMID: 12668152

[ref96] Patnala R. Arumugam T. V. Gupta N. Dheen S. T. (2017). HDAC inhibitor sodium butyrate-mediated epigenetic regulation enhances neuroprotective function of microglia during ischemic stroke. Mol. Neurobiol. 54, 6391–6411. doi: 10.1007/s12035-016-0149-z, PMID: 27722928

[ref97] Pereira C. R. Criado M. B. Machado J. Pereira C. T. Santos M. J. (2021). Acute effects of acupuncture in balance and gait of Parkinson disease patients - a preliminary study. Complement. Ther. Clin. Pract. 45:101479. doi: 10.1016/j.ctcp.2021.101479, PMID: 34543873

[ref98] Pourhamzeh M. Moravej F. G. Arabi M. Shahriari E. Mehrabi S. Ward R.. (2022). The roles of serotonin in neuropsychiatric disorders. Cell. Mol. Neurobiol. 42, 1671–1692. doi: 10.1007/s10571-021-01064-9, PMID: 33651238 PMC11421740

[ref99] Qin X.-Y. Zhang S.-P. Cao C. Loh Y. P. Cheng Y. (2016). Aberrations in peripheral inflammatory cytokine levels in Parkinson disease: a systematic review and meta-analysis. JAMA Neurol. 73, 1316–1324. doi: 10.1001/jamaneurol.2016.2742, PMID: 27668667

[ref100] Rich B. E. Jackson J. C. de Ora L. O. Long Z. G. Uyeda K. S. Bess E. N. (2022). Alternative pathway for dopamine production by acetogenic gut bacteria that O-demethylate 3-methoxytyramine, a metabolite of catechol O-methyltransferase. J. Appl. Microbiol. 133, 1697–1708. doi: 10.1111/jam.15682, PMID: 35737746 PMC9544265

[ref101] Romano S. Savva G. M. Bedarf J. R. Charles I. G. Hildebrand F. Narbad A. (2021). Meta-analysis of the Parkinson’s disease gut microbiome suggests alterations linked to intestinal inflammation. Npj Parkinsons Dis. 7:27. doi: 10.1038/s41531-021-00156-z, PMID: 33692356 PMC7946946

[ref102] Sampson T. R. Challis C. Jain N. Moiseyenko A. Ladinsky M. S. Shastri G. G.. (2020). A gut bacterial amyloid promotes α-synuclein aggregation and motor impairment in mice. eLife 9:e53111. doi: 10.7554/eLife.53111, PMID: 32043464 PMC7012599

[ref103] Sancho-Alonso M. Sarriés-Serrano U. Miquel-Rio L. Yanes Castilla C. Paz V. Meana J. J.. (2024). New insights into the effects of serotonin on Parkinson’s disease and depression through its role in the gastrointestinal tract. Span. J. Psychiatry Ment. Health 24, S2950–S2853. doi: 10.1016/j.sjpmh.2024.07.002, PMID: 38992345

[ref104] Sandler M. Goodwin B. L. Ruthven C. R. (1971). Therapeutic implications in Parkinsonism of m-tyramine formation from L-dopa in man. Nature 229, 414–416. doi: 10.1038/229414a0 4926994

[ref105] Seguella L. Sarnelli G. Esposito G. (2020). Leaky gut, dysbiosis, and enteric glia activation: the trilogy behind the intestinal origin of Parkinson’s disease. Neural Regen. Res. 15, 1037–1038. doi: 10.4103/1673-5374.270308, PMID: 31823880 PMC7034261

[ref106] Shah M. Deeb J. Fernando M. Noyce A. Visentin E. Findley L. J.. (2009). Abnormality of taste and smell in Parkinson’s disease. Park. Relat. Disord. 15, 232–237. doi: 10.1016/j.parkreldis.2008.05.008, PMID: 18606556

[ref107] Sharma S. Taliyan R. Singh S. (2015). Beneficial effects of sodium butyrate in 6-OHDA induced neurotoxicity and behavioral abnormalities: modulation of histone deacetylase activity. Behav. Brain Res. 291, 306–314. doi: 10.1016/j.bbr.2015.05.052, PMID: 26048426

[ref108] Shen T. Yue Y. He T. Huang C. Qu B. Lv W.. (2021). The association between the gut microbiota and Parkinson’s disease, a meta-analysis. Front. Aging Neurosci. 13:636545. doi: 10.3389/fnagi.2021.636545, PMID: 33643026 PMC7907649

[ref109] Sheng L. Stewart T. Yang D. Thorland E. Soltys D. Aro P.. (2020). Erythrocytic α-synuclein contained in microvesicles regulates astrocytic glutamate homeostasis: a new perspective on Parkinson’s disease pathogenesis. Acta Neuropathol. Commun. 8:102. doi: 10.1186/s40478-020-00983-w, PMID: 32641150 PMC7346449

[ref110] Soliman M. L. Combs C. K. Rosenberger T. A. (2013). Modulation of inflammatory cytokines and mitogen-activated protein kinases by acetate in primary astrocytes. J. Neuroimmune Pharmacol.: Off J. Soc. Neuroimmune Pharmacol. 8, 287–300. doi: 10.1007/s11481-012-9426-4, PMID: 23233245 PMC3587660

[ref111] Soliman M. L. Puig K. L. Combs C. K. Rosenberger T. A. (2012). Acetate reduces microglia inflammatory signaling in vitro. J. Neurochem. 123, 555–567. doi: 10.1111/j.1471-4159.2012.07955.x, PMID: 22924711 PMC3472042

[ref112] Sun J. Wang F. Hong G. Pang M. Xu H. Li H.. (2016). Antidepressant-like effects of sodium butyrate and its possible mechanisms of action in mice exposed to chronic unpredictable mild stress. Neurosci. Lett. 618, 159–166. doi: 10.1016/j.neulet.2016.03.003, PMID: 26957230

[ref113] Sung H.-Y. Park J.-W. Kim J.-S. (2014). The frequency and severity of gastrointestinal symptoms in patients with early Parkinson’s disease. J. Mov. Disord. 7, 7–12. doi: 10.14802/jmd.14002, PMID: 24926404 PMC4051727

[ref114] Tan W. Xie F. Zhou J. Pan Z. Liao M. Zhuang L. (2024). Efficacy and safety of acupuncture therapy for neuropsychiatric symptoms among patients with Parkinson’s disease: a systematic review and meta-analysis. Clin. Rehabil. 38, 1044–1062. doi: 10.1177/02692155241258278, PMID: 38840478 PMC11348633

[ref115] Techaniyom P. Korsirikoon C. Rungruang T. Pakaprot N. Prombutara P. Mukda S.. (2024). Cold-pressed perilla seed oil: investigating its protective influence on the gut-brain axis in mice with rotenone-induced Parkinson’s disease. Food Sci. Nutr. 12, 6259–6283. doi: 10.1002/fsn3.4265, PMID: 39554352 PMC11561828

[ref116] Tolosa E. Garrido A. Scholz S. W. (2021). Challenges in the diagnosis of Parkinson’s disease. Lancet Neurol. 20, 385–397. doi: 10.1016/S1474-4422(21)00030-2, PMID: 33894193 PMC8185633

[ref117] Tsai C. Y. Liao W. L. Wu H. M. Chang C. W. Chen W. L. Hsieh C. L. (2024). Acupuncture improves neurological function and anti-inflammatory effect in patients with acute ischemic stroke: a double-blinded randomized controlled trial. Complement. Ther. Med. 82:103049. doi: 10.1016/j.ctim.2024.103049, PMID: 38729273

[ref118] Vicentini F. A. Keenan C. M. Wallace L. E. Woods C. Cavin J.-B. Flockton A. R.. (2021). Intestinal microbiota shapes gut physiology and regulates enteric neurons and glia. Microbiome 9:210. doi: 10.1186/s40168-021-01165-z, PMID: 34702353 PMC8549243

[ref119] Vilela C. Araújo B. Soares-Guedes C. Caridade-Silva R. Martins-Macedo J. Teixeira C.. (2024). From the gut to the brain: is microbiota a new paradigm in Parkinson’s disease treatment? Cells 13:770. doi: 10.3390/cells13090770, PMID: 38727306 PMC11083070

[ref120] Wang J. Y. Chen J.-Y. Lian Y.-Z. Zhang W.-Y. Wang M.-Y. Liu R.-P.. (2023). Reduced platelet 5-HT content is associated with rest tremor in Parkinson’s disease. Park. Relat. Disord. 108:105314. doi: 10.1016/j.parkreldis.2023.105314, PMID: 36739793

[ref121] Wang Y. Tong Q. Ma S.-R. Zhao Z.-X. Pan L.-B. Cong L.. (2021). Oral berberine improves brain dopa/dopamine levels to ameliorate Parkinson’s disease by regulating gut microbiota. Signal Transduct. Target. Ther. 6:77. doi: 10.1038/s41392-020-00456-5, PMID: 33623004 PMC7902645

[ref122] Wang L. Wang Z. Lan Y. Tuo Y. Ma S. Liu X. (2023). Inulin attenuates blood-brain barrier permeability and alleviates behavioral disorders by modulating the TLR4/MyD88/NF-κB pathway in mice with chronic stress. J. Agric. Food Chem. 71, 13325–13337. doi: 10.1021/acs.jafc.3c03568, PMID: 37642581

[ref123] Wang H. Wang Q. Liang C. Pan L. Hu H. Fang H. (2023). Acupuncture improved hepatic steatosis in HFD-induced NAFLD rats by regulating intestinal microbiota. Front. Microbiol. 14:1131092. doi: 10.3389/fmicb.2023.1131092, PMID: 37007509 PMC10061080

[ref124] Wang C. Yang M. Liu D. Zheng C. (2024). Metabolic rescue of α-synuclein-induced neurodegeneration through propionate supplementation and intestine-neuron signaling in *C. elegans*. Cell Rep. 43:113865. doi: 10.1016/j.celrep.2024.113865, PMID: 38412096

[ref125] Wang P. Zhang Y. Gong Y. Yang R. Chen Z. Hu W.. (2018). Sodium butyrate triggers a functional elongation of microglial process via akt-small RhoGTPase activation and HDACs inhibition. Neurobiol. Dis. 111, 12–25. doi: 10.1016/j.nbd.2017.12.006, PMID: 29248540

[ref126] Warren A. Nyavor Y. Zarabian N. Mahoney A. Frame L. A. (2024). The microbiota-gut-brain-immune interface in the pathogenesis of neuroinflammatory diseases: a narrative review of the emerging literature. Front. Immunol. 15:1365673. doi: 10.3389/fimmu.2024.1365673, PMID: 38817603 PMC11137262

[ref127] Wen X. Li K. Wen H. Wang Q. Wu Z. Yao X.. (2021). Acupuncture-related therapies for Parkinson’s disease: a meta-analysis and qualitative review. Front. Aging Neurosci. 13:676827. doi: 10.3389/fnagi.2021.676827, PMID: 34276340 PMC8282198

[ref128] Wu G. Jiang Z. Pu Y. Chen S. Wang T. Wang Y.. (2022). Serum short-chain fatty acids and its correlation with motor and non-motor symptoms in Parkinson’s disease patients. BMC Neurol. 22:13. doi: 10.1186/s12883-021-02544-7, PMID: 34996385 PMC8740341

[ref129] Wu Y. Kong Q. Li Y. Feng Y. Zhang B. Liu Y.. (2025). Potential scalp acupuncture and brain stimulation targets for common neurological disorders: evidence from neuroimaging studies. Chin. Med. 20:58. doi: 10.1186/s13020-025-01106-0, PMID: 40329319 PMC12057072

[ref130] Wu J. Li C.-S. Huang W.-Y. Zhou S.-Y. Zhao L.-P. Li T.. (2025). Gut microbiota promote the propagation of pathologic α-syn from gut to brain in a gut-originated mouse model of Parkinson’s disease. Brain Behav. Immun. 128, 152–169. doi: 10.1016/j.bbi.2025.04.001, PMID: 40187668

[ref131] Wu J. Wang Y. Wang X. Xie Y. Li W. (2023). A systematic review and meta-analysis of acupuncture in Parkinson’s disease with dysphagia. Front. Neurol. 14:1099012. doi: 10.3389/fneur.2023.1099012, PMID: 37305760 PMC10251408

[ref132] Xu K. Wang S. Bai Y. Wang Y.-L. Li Y. Zhang C.-G.. (2025). Research progress of acupuncture regulating enteric nervous system in Parkinson’s disease. Zhen Ci Yan Jiu 50, 110–115. doi: 10.13702/j.1000-0607.20230775 39961766

[ref133] Xue H. He H.-X. Wu D. Fan W.-H. Li Y.-X. (2024). An overview of systematic reviews of acupuncture for Parkinson’s disease. Front. Neurosci. 18:1415008. doi: 10.3389/fnins.2024.1415008, PMID: 39280262 PMC11392918

[ref134] Yan M. Fan J. Liu X. Li Y. Wang Y. Tan W.. (2024). Acupuncture and sleep quality among patients with Parkinson disease: a randomized clinical trial. JAMA Netw. Open 7:e2417862. doi: 10.1001/jamanetworkopen.2024.17862, PMID: 38922617 PMC11208974

[ref135] Yano J. M. Yu K. Donaldson G. P. Shastri G. G. Ann P. Ma L.. (2015). Indigenous bacteria from the gut microbiota regulate host serotonin biosynthesis. Cell 161, 264–276. doi: 10.1016/j.cell.2015.02.047, PMID: 25860609 PMC4393509

[ref136] Yeo S. Song J. Lim S. (2020). Acupuncture inhibits the increase in alpha-synuclein in substantia nigra in an MPTP-induced Parkinsonism mouse model. Adv. Exp. Med. Biol. 1232, 401–408. doi: 10.1007/978-3-030-34461-0_51 31893437

[ref137] Yu J. Meng J. Qin Z. Yu Y. Liang Y. Wang Y.. (2023). Dysbiosis of gut microbiota inhibits NMNAT2 to promote neurobehavioral deficits and oxidative stress response in the 6-OHDA-lesioned rat model of Parkinson’s disease. J. Neuroinflammation 20:117. doi: 10.1186/s12974-023-02782-1, PMID: 37208728 PMC10199500

[ref138] Zeng H.-J. Zhao W.-J. Luo P.-C. Zhang X.-Y. Luo S.-Y. Li Y.. (2025). Acupuncture therapy on dysphagia in patients with Parkinson’s disease: a randomized controlled study. Chin. J. Integr. Med. 31, 261–269. doi: 10.1007/s11655-024-3668-x, PMID: 39305459

[ref139] Zhang H. Cao X.-Y. Wang L.-N. Tong Q. Sun H.-M. Gan C.-T.. (2023). Transcutaneous auricular vagus nerve stimulation improves gait and cortical activity in Parkinson’s disease: a pilot randomized study. CNS Neurosci. Ther. 29, 3889–3900. doi: 10.1111/cns.14309, PMID: 37311693 PMC10651956

[ref140] Zhang C. Chen T. Fan M. Tian J. Zhang S. Zhao Z.. (2024). Electroacupuncture improves gastrointestinal motility through a central-cholinergic pathway-mediated GDNF releasing from intestinal glial cells to protect intestinal neurons in Parkinson’s disease rats. Neurother.: J. Am. Soc. Exp. Neurother. 21:e00369. doi: 10.1016/j.neurot.2024.e00369, PMID: 38744625 PMC11305299

[ref141] Zhao L. Niu Q. Yang K. Zhao K. Shao Y. Zhu F. (2024). Acupuncture for constipation in Parkinson’s disease: a systematic review and meta-analysis of randomized controlled trials. Medicine (Baltimore) 103:e38937. doi: 10.1097/MD.0000000000038937, PMID: 39029044 PMC11398760

[ref142] Zhu M. Liu X. Ye Y. Yan X. Cheng Y. Zhao L.. (2022). Gut microbiota: a novel therapeutic target for Parkinson’s disease. Front. Immunol. 13:937555. doi: 10.3389/fimmu.2022.937555, PMID: 35812394 PMC9263276

[ref143] Zou Y. Huang T. Pang A. Zhou H. Geng X. (2024). Electroacupuncture regulates glucose metabolism by inhibiting SGLT1 levels, inhibiting microglial polarization, and alleviating Parkinson’s disease. Exp. Gerontol. 196:112558. doi: 10.1016/j.exger.2024.112558, PMID: 39197673

